# Cytotoxicity of Endoperoxides from the Caribbean Sponge *Plakortis halichondrioides* towards Sensitive and Multidrug-Resistant Leukemia Cells: Acids vs. Esters Activity Evaluation

**DOI:** 10.3390/md15030063

**Published:** 2017-03-03

**Authors:** Tanja Schirmeister, Swarna Oli, Hongmei Wu, Gerardo Della Sala, Valeria Costantino, Ean-Jeong Seo, Thomas Efferth

**Affiliations:** 1 Institute of Pharmacy and Biochemistry, Johannes Gutenberg University Mainz, Staudinger Weg 5, 55128 Mainz, Germany; swarnoli@uni-mainz.de (S.O.); hongmei@uni-mainz.de (H.W.); seo@uni-mainz.de (E.-J.S.); efferth@uni-mainz.de (T.E.); 2 The NeaNat Group, Dipartimento di Farmacia, Università degli Studi di Napoli Federico II, via D. Montesano 49, 80131 Napoli, Italy; gerardo.dellasala@unina.it (G.D.S.); valeria.costantino@unina.it (V.C.)

**Keywords:** Caribbean sponge, plakortide, endoperoxide, leukemia, multi-drug resistant leukemia, cytotoxicity

## Abstract

The 6-epimer of the plakortide H acid (**1**), along with the endoperoxides plakortide E (**2**), plakortin (**3**), and dihydroplakortin (**4**) have been isolated from a sample of the Caribbean sponge *Plakortis halichondrioides*. To perform a comparative study on the cytotoxicity towards the drug-sensitive leukemia CCRF-CEM cell line and its multi-drug resistant subline CEM/ADR5000, the acid of plakortin, namely plakortic acid (**5**), as well as the esters plakortide E methyl ester (**6**) and 6-epi-plakortide H (**7**) were synthesized by hydrolysis and Steglich esterification, respectively. The data obtained showed that the acids (**1**, **2**, **5**) exhibited potent cytotoxicity towards both cell lines, whereas the esters showed no activity (**6**, **7**) or weaker activity (**3**, **4**) compared to their corresponding acids. Plakortic acid (**5**) was the most promising derivative with half maximal inhibitory concentration (IC_50)_ values of ca. 0.20 µM for both cell lines.

## 1. Introduction

**Figure 1:**
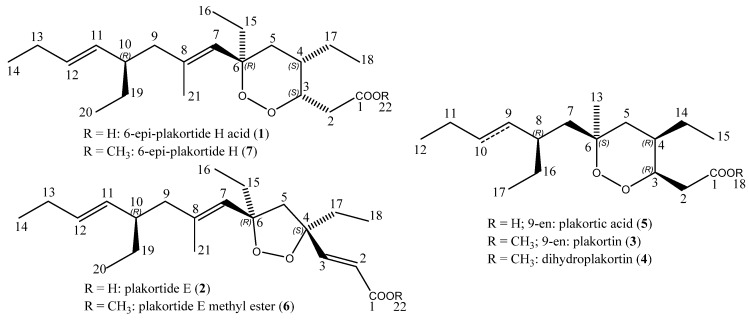
Structures of natural (**1**, **2**, **3**, **4**) and semi-synthetic (**5**, **6**, **7**) endoperoxides from a sample of the sponge *Plakortis halichondrioides*: 6-epi-plakortide H acid (**1**), plakortide E (**2**), plakortin (**3**), dihydroplakortin (**4**), plakortic acid (**5**), plakortide E methyl ester (**6**), and 6-epi-plakortide H (**7**).

Marine organisms are excellent sources of novel skeletons ranging from small terpene molecules [1,2], mixed polyketide-peptide biogenesis [3,4], to more complex carbohydrate-based scaffolds [5,6]. Many of these novel skeletons [7] have been tested for their possible role as lead compounds in the search for new drugs for various diseases. Among the different classes, endoperoxides such as the famous artemisinin from *Artemisia annua* L. are well-known for their bioactivity. The Chinese scientist Youyou Tu isolated artemisinin and described its antimalarial activity in the 1970s. She was honoured with the Nobel Prize for Physiology or Medicine in 2015 [8]. Artemisinin and its derivatives are also active against various cancer cell lines, especially against leukemia and colon cancer [9,10]. The first long-term treatment of cancer patients with artesunate in combination with standard chemotherapy has been described [11]. In 2009, the combined effects of artesunate and rituximab on malignant B-cells were reported [12]. Clinical pilot phase I/II trials in veterinary tumors and human cancer patients demonstrated that the artemisinin derivative artesunate possesses clinical anticancer activity at tolerable side effects [13,14,15]. It can be speculated that not only artemisinin-type drugs, but also other endoperoxides may reveal anticancer activity. This hypothesis is substantiated by reports on the cytotoxicity of natural and synthetic endoperoxides towards tumor cell lines [16,17,18,19,20,21,22,23,24,25]. Endoperoxides are, therefore, worth investigating to unravel their full potential as anticancer drug leads. The Caribbean sponge *Plakortis halichondrioides* produces endoperoxides which were assumed to be synthesized by the polyketide pathway [26,27]. Similar to artesunate, these metabolites did not only display antimalarial activity, but also cytotoxic activity against several tumor cell lines [28,29,30]. From a sample of this sponge, we isolated plakortide E (**2**, Figure 1) and found that it was also active against trypanosomes [31]. Here, we report the cytotoxicity towards the drug-sensitive leukemia CCRF-CEM cell line (human Caucasian acute lymphoblastic leukemia, childhood T acute lymphoblastic leukemia) and its multi-drug resistant subline CEM/ADR5000 (multi-drug resistant CCRF cell line) (Table 2), of seven derivatives (Figure 1): the 6-epimer of the plakortide H acid (**1**) [32,33] along with the endoperoxides plakortide E (**2**), plakortin (**3**) [34,35,36], and dihydroplakortin (**4**) [36,37] that have been isolated from a sample of the Caribbean sponge *Plakortis halichondrioides*. In addition, the acid of plakortin, namely plakortic acid (**5**) [38,39], as well as the esters plakortide E methyl ester (**6**) [40,41] and the ester 6-epi-plakortide H (**7**) were synthesized by hydrolysis (plakortic acid) and Steglich esterification (plakortide E methyl ester and 6-epi-plakortide H), respectively, to perform a comparative study. There are some discrepancies within the literature concerning the nomenclature of plakortides and their esters: According to reference [22] plakortide I is the acid of the methyl ester plakortide H. Also reference [27] and the reference [32] term the methyl ester plakortide H. In contrast, the reference [38] describes plakortide H as the respective acid and plakortide I as its 11,12-dihydro derivative. In the present manuscript, we refer to plakortide H as the methyl ester, and accordingly compound **1** is the 6-epimer of plakortide H acid, and compound **7** the 6-epimer of plakortide H. There are also discrepancies concerning the structure of plakortic acid: According to reference [20] the natural compound named plakortic acid is rather an epoxide than an endoperoxide. Reference [38] in contrast assigns the structure of the acid of plakortin to plakortic acid. In the present manuscript, we refer to plakortic acid **5** as the acid of plakortin **3**.

## 2. Results

### 2.1. Isolation, Semi-Syntheses, and Identification of 6-Epi-Plakortide H Acid *(**1**)* and Its Methyl Ester 6-Epi-Plakortide H *(**7**)*

**Figure 2:**
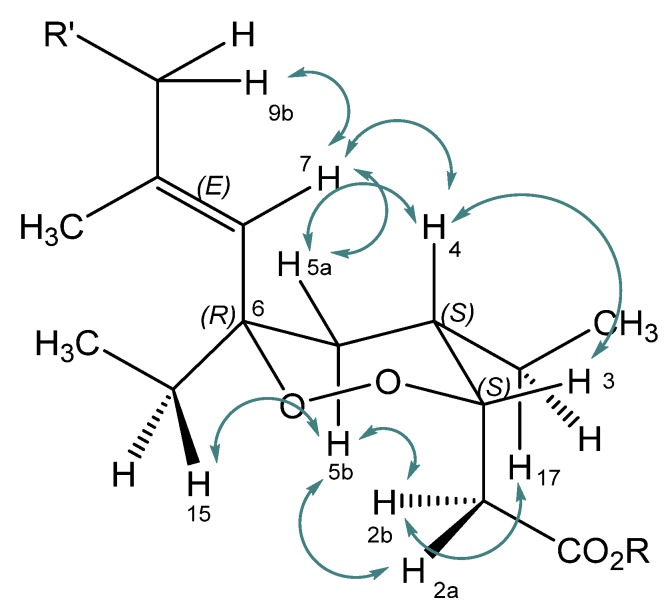
Selected nuclear Overhauser effect (NOE) correlations observed for 6-epi-plakortide H acid (**1**, *R* = H) and the methyl ester 6-epi-plakortide H (**7**, *R* = CH_3_).

A sample of the sponge *Plakortis halichondrioides*, order Homosclerophorida, family Plakinidae, (640 g freeze-dried) was collected via scuba diving along the coast of Inagua Island (GPS coordinates 21°10.7684’ N 73°9.1608’ W) on 7 July 2013 at a depth of 30 m. After collection, the sample was unambiguously identified on board using a web-based photographic and taxonomic key [42]. The sample was immediately frozen and stored. A voucher sample with the reference no. 13/7/13 has been deposited at the Dipartimento di Farmacia, Università degli Studi Napoli “Federico II”. For this study, the sponge tissue was cut into small pieces, lyophilized, and then sequentially extracted with cyclohexane, methylene chloride, and methanol solvents. The crude methylene chloride extract was subjected to column chromatography using a gradient solvent system starting with cyclohexane and changing gradually to methylene chloride, chloroform, and finally to methanol. Based upon thin layer chromatography (TLC) analysis the fractions were combined to yield six fractions I–VI (I-3.2 g, II-5.1 g, III-2.8 g, IV-4.3 g, V-6.9 g, VI-7.5 g). The fraction IV was subjected to preparative reversed-phase high performance liquid chromatography (RP-HPLC) chromatography to yield a fraction (termed **1mix**, 0.5005 g), which was identified as a mixture of several acidic compounds. The fraction was converted into an ester mixture (termed **7mix**) using the Steglich esterification procedure with methanol, dicyclohexylcarbodiimide (DCC), and 4-dimethylamino pyridine (DMAP). Then, the mixture of esters was purified using preparative RP-HPLC to yield a pure methyl ester (**7**), which eluted at 14 min as a pale yellow viscous oil. The ester which was later on identified as the methyl ester derivative of the 6-epi-plakortide H acid was hydrolyzed in THF/water (4:1; 10 mL) with LiOH (3 eq.). The residue obtained after acidic workup was further purified via preparative RP-HPLC to yield the pure acid (**1**). The structure of the compound was analyzed by ^1^H, ^13^C, correlation spectroscopy (COSY), and nuclear Overhauser exchange spectroscopy (NOESY) nuclear magnetic resonance (NMR) spectroscopy and mass spectrometry and, according to the literature [29,33] and NOESY data, the pure acid was identified as a diastereomer of plakortide H acid, namely the 6-epimer. In fact, the NOESY data and coupling constants are in agreement with those found for plakortides M and N [29] and are in agreement with the literature [33] and thus, the same configuration was also assumed for the isolated compound, namely the (*R*)-configuration at the 6-position and the (*R*)-configuration at the carbon atom 10 [33]. For coupling of H-3 (equatorial, eq.) and H-4(axial, ax.) a constant of *J* = 5.2 Hz was found. NOESY correlations (Figure 2) were observed between H-2 and H-5b(ax.), H-3(eq.) and H-4(ax.), H-4(ax.) and H-5a(eq.), H-4(ax.) and H-7, H5a(eq.) and H-7, and H-5b(ax.) and H-15 (Figure 2). This is only possible with an equatorial position of the ethyl moiety (i.e., (*R*)-configuration) at C-6. Thus, the absolute configuration was assigned as (6*R*,10*R*). NMR data for compound (**1**) are reported in Table 1, and the NMR data of methyl ester (**7**) are reported in the Supplementary Materials.

### 2.2. Isolation and Identification of Plakortide E *(**2**)*, Plakortin *(**3**)*, and Dihydroplakortin *(**4**)*

The crude cyclohexane extract was subjected to chromatography on silica gel using the isocratic solvent cyclohexane/methylene chloride/methanol/formic acid (2:1:1:0.5). Based on TLC analysis, the eluted fractions were combined to yield five fractions, named I–V (I-1.405 g, II-1.77 g, III-7.18 g, IV-3.45 g, V-1.07 g). Fraction III was subjected to column chromatography on silica gel using a gradient solvent system starting with cyclohexane/methylene dichloride 90:10 and successively changing to chloroform/methanol 10:90 providing seven sub-fractions, named A–G (A-0.532 g, B-0.6912 g, C-0.8149 g, D-1.063 g, E-0.1401 g, F-2.8811 g, G-0.2108 g). Sub-fraction E was purified by preparative RP-HPLC (Phenomenex Hyperclone 5 µ) using the mobile phase methanol/water 70:30 containing 0.1% formic acid (flow 8 mL/min). Plakortide E (**2**) (Figure 1) eluted at a retention time of 40 min. The NMR (^1^H, ^13^C, 2D NMR) and mass spectrometry (MS) data and the optical rotation were in agreement with those reported previously [31]. Subfraction D was purified by preparative RP-HPLC (Phenomenex Hyperclone 5 µ) using methanol/acetonitrile/water 73:6:21 containing 0.1% formic acid as the mobile phase (flow 9 mL/min). Plakortin (**3**) eluted at a retention time of 18 min. The structure of the compound was analyzed by NMR spectroscopy and mass spectrometry, and according to literature data [28,34,36], the compound was identified as plakortin. Sub-fraction C was purified by semi-preparative RP-HPLC (Phenomenex Hyperclone 5 µ) using acetonitrile/water 60:30 containing 0.1% formic acid as the mobile phase (flow 2 mL/min). Dihydroplakortin (**4**) eluted at a retention time of 41 min. The structure of the compound was elucidated by NMR spectroscopy, mass spectrometry, and optical rotation, and was assigned according to the literature data [37] as dihydroplakortin. The NMR data of the compounds are presented in the Supplementary Materials.

### 2.3. Semi-Synthesis of Plakortic Acid *(**5**)* and Plakortide E Methyl Ester *(**6**)*

Plakortin (**3**) was converted into its acid, plakortic acid (**5**), by hydrolysis with LiOH (3 eq.) in THF/water (4:1). After acidic work-up, the residue was further purified via preparative RP-HPLC. The structure of the compound was analyzed by NMR spectroscopy, mass spectrometry, and optical rotation, and, according to the literature data [39], the compound was identified as plakortic acid. Plakortide E (**2**) was converted into its ester (**6**) via Steglich esterification with methanol, DCC, and DMAP. The raw product was further purified via preparative RP-HPLC. The NMR data were in agreement with the literature data [40,41]. The NMR data of the compounds are reported in the Supplementary Materials.

### 2.4. Cytotoxicity Assay

Drug-sensitive leukemia CCRF-CEM cells and its multi-drug resistant (MDR) subline CEM/ADR5000 were used to test the cytotoxicity of endoperoxides **1**–**7**. The resazurin reduction assay [43] was performed to determine the cytotoxicity of the seven compounds in a concentration range of 0.001 to 10 µg/mL as previously described [44,45,46,47,48]. Cytotoxicity of established cytostatic drugs against sensitive and multi-drug resistant leukemia cell lines was previously reported by our group (Table 2) [49]. The IC_50_ values were determined from dose response curves and resistance ratios were calculated by dividing the IC_50_ of resistant cells by the IC_50_ of the corresponding parental cells. A degree of resistance >1 indicated that the compound kills the parental cells more effectively than the MDR cells, indicating cross-resistance, while a degree of resistance <1 indicates that the drug kills the MDR cells more effectively, indicating hypersensitivity (collateral sensitivity). The results are shown in Table 2.

## 3. Discussion

The most obvious structure-activity relationship (SAR) concerns the esters **6**, **7**, and their acid counterparts **2** and **1**: the free acids possessed cytotoxic activity at micromolar concentrations, while the relevant esters were inactive. Similarly, plakortic acid (**5**) was more potent (about 10-fold) than its natural ester plakortin (**3**). Moreover, the side chain did not have any influence on the cytotoxicity (compare **1** and **5**). In contrast, the size of the endoperoxide ring (five-membered vs. six-membered) was important, with the six-membered 6-epi-plakortide H acid (**1**) being 10-fold more active than the five-membered endoperoxide plakortide E (**2**) with the same side chain. Plakortide E (**2**) and its methyl ester (**6**) also possess a double bond activated by an electron-withdrawing substituent (acid or ester) for nucleophilic attack [50], which might also contribute to cytotoxicity. However, the data did not support this assumption, since the methyl ester of plakortide E (**6**) which also contains the activated double bond was inactive.

The inactivity or lower activity of the ester derivatives compared to their acid counterparts was in line with previous findings. For the plakortide H acid and its methyl ester, high cytotoxic effects (IC_50_ <0.7 µg/mL) and inactivity (>100 µg/mL), respectively, were found against the cell lines NIH3T3 (mouse embryo fibroblast), SSVNIH3T3 (Simian sarcoma virus-transformed NIH3T3), and KA3IT (virally transformed NIH3T3) [28]. Cytotoxic activity against tumor cells (including CCRF-CEM) was also reported for the acids plakortide M and N [29]. On the other hand, plakortide F as the methyl ester with a six-membered endoperoxide structure showed some activity against cancer cell lines [51]. Taking into account the facile hydrolysis of methyl esters in vivo but also within cells, the question arises whether the cytotoxic activity of these esters could be attributed at least in part to their acid forms. For the activity in vivo, the methyl esters might be more favourable due to better membrane permeability properties and oral availability compared to the acids. Furthermore, they may act as typical ester pro-drugs.

The degree of resistance of the seven compounds was >1 in all cases, i.e., compounds were more effective against the sensitive cells than against the resistant cells. Plakortic acid (**5**), with comparable IC_50_ values for both cell lines (0.19 µM and 0.24 µM for the sensitive and resistant cells, respectively) seems to be the most promising derivative, since it was highly potent and the resistance ratio was still around 1. However, owing to the fact that CEM/ADR5000 reveal high degrees of cross-resistance (in the range of hundreds to thousands) to standard drugs such as doxorubicin, daunorubicin, vincristine, vinblastine, paclitaxel, docetaxel, and others (Table 2) [49], it is well justified that compounds with degrees of resistance below or around two can be considered as being active against multidrug-resistant cells. In light of better pharmacokinetic properties, the ester derivative plakortin (**3**), which is not as active but displays a similar resistance ratio, may even be the better candidate for further evaluation.

In summary, we present the cytotoxic properties of several plakortide acids and esters. The SAR studies confirmed that the cytotoxic activity is related to the peroxide function as previously shown [52]. In addition, we found that it is also related to the chemical properties of the acid group, versus the ester. Further evaluations will therefore address this question in more detail.

## 4. Materials and Methods

General Experimental Procedures. Optical rotations were measured with a Krüss Optronic GmbH polarimeter (Hamburg, Germany). ^1^H spectral data were generated with a Bruker Fourier 300 (300 MHz) and Bruker Avance III 600 (600 MHz, 5 mm TCI-CryoProbe with z-gradient and ATM, SampleXPress Lite 16 sample changer) FT-NMR spectrometer (Karlsruhe, Germany), and the ^13^C spectral data, COSY, NOESY, DEPT (distortionless enhancement by polarization transfer, HMQC (heteronuclear multiple-quantum correlation), and HMBC (heteronuclear multiple bond correlation) experiments were measured with the 600 MHz Bruker Avance III 600 FT-NMR spectrometer (Karlsruhe, Germany). MS were carried out with a Bruker micrOTOF 88 mass spectrometer (Bremen, Germany) and a LC/MSD-Trap-Mass spectrometer (Agilent Technologies, LC/MSD Ion Trap, Waldbronn, Germany). Column chromatography was performed on silica gel (0.063–0.200 mm mesh, Merck, Darmstadt, Germany). TLC analyses were carried out using pre-coated silica gel 60 F254 plates (0.20 mm, Merck), and spots were visualized using vanillin spray reagent. DCC, DMAP, and reagents were purchased from Sigma-Aldrich (Munich, Germany) or Fluka (Munich, Germany). Solvents were purchased from Roth (Karlsruhe, Germany) or Merck. High performance liquid chromatography was performed on a Varian ProStar analytical/preparative HPLC Linear Upscale system (0.05–50 mL/min at 275 bar pressure with scale-mast), a preparative autosampler and a 2-channel UV detector (Waldbronn, Germany). The detection wavelengths were 254 nm and 230 nm.

### 4.1. 6-Epi-plakortide H acid *(**1**)*, [[(3S,4S,6R)-4,6-Diethyl-6-((1E,5E)-4-(R)-ethyl-2-methyl-octa-1,5-dienyl)-[1,2]dioxan-3-yl]-acetic acid]

The methyl ester (**7**) was hydrolysed using the method described below for plakortic acid (**5**). The residue was purified using preparative RP-HPLC. Yellow viscous oil (4.1 mg); ${\left[\alpha \right]}_{D}^{23}$ = −157.84 (*c* 0.0037, CHCl_3_) (reference [33] reports ${\left[\alpha \right]}_{D}^{20}$ = −145 (*c* 1.1, CHCl_3_)); ESI-MS: *m/z* 375.25 [M + Na]^+^, calcd. for C_21_H_36_O_4_, 352.51. NMR data are reported in Table 1; since they were found to be identical to those described in reference [33], the compound was identified as the 6-epimer of plakortide H acid.

Plakortide E (**2**): 18 mg; the ${\left[\alpha \right]}_{D}^{23}$, ^1^H and ^13^C NMR, and MS data were identical in all respects to those previously reported in the literature [31].

Plakortin (**3**): pale yellow coloured oil (49.8 mg); ${\left[\alpha \right]}_{D}^{23}$ = +154.93 (*c* 0.0075, CHCl_3_); [53] (see in the reference ${\left[\alpha \right]}_{D}^{20}$ = +189 (*c* 2.9, CHCl_3_)) LC-MS: *m/z* 334.6 [M + Na]^+^, calcd. for C_18_H_32_O_4_ *m/z* 312.44; ^1^H and ^13^C NMR data were identical in all respects to those previously reported in the literature [28,34,35].

Dihydroplakortin (**4**): colourless oil (1.8 mg); ESI-MS: *m/z* 337.20 [M + Na]^+^, calcd. for C_18_H_34_O_4_ *m/z* 314.46; the optical rotation [53] (see in the reference ${\left[\alpha \right]}_{D}^{20}$ = +49 (*c* 0.002, CHCl_3_)) was not determined due to insufficient quantity of the substance. ^1^H and ^13^C NMR data were identical in all respects to those previously reported in the literature [37].

Plakortic acid (**5**): Plakortin (**3**) was converted into its acid, plakortic acid, by hydrolysis. To a solution of plakortin (43.2 mg, 0.138 mmol) in THF/H_2_O (4:1; 10 mL), LiOH (17.4 mg, 3 eq.) was added at 0 °C. The reaction mixture was allowed to warm to room temperature and allowed to stir for 24 h. The reaction was monitored using TLC until the starting material disappeared. Then the reaction mixture was acidified to pH 2 with 10% aqueous HCl and extracted with ether (3 × 10 mL). The combined extracts were washed with NaCl solution (15 mL) and dried over anhydrous Na_2_SO_4_. The residue was further purified via preparative RP-HPLC. Colourless oil (4.1 mg), ${\left[\alpha \right]}_{D}^{23}$ = +109 (*c* 0.002, CHCl_3_); LC-MS: *m/z* 321.2 [M + Na]^+^, calcd. for C_17_H_30_O_4_ *m/z* 298.42. ^1^H and ^13^C NMR data were identical in all respects to those previously reported in the literature [39].

Plakortide E methyl ester (**6**): Plakortide E (**2**) was converted into its ester form via Steglich esterification. To a solution of plakortide E (9.6 mg, 0.0274 mmol in dichloromethane at 0 °C), methanol (0.88 mL, 0.4314 mmol, 1.0 eq.) was first added; then, 1.05 eq. DCC (6.01 mg, 0.0291 mmol) and 0.1 eq. DMAP (0.5 mg, 0.0041 mmol) were added. The reaction mixture was stirred for 1 h at 0 °C and then at room temperature for 24 h. The colourless solid by-product *N*,*N*′-dicyclohexylurea was filtered off and the organic phase was washed with half-saturated solutions of ammonium chloride, sodium bicarbonate, and sodium chloride. It was then dried over sodium sulphate, filtered off, and the organic phase was removed in vacuo. The raw product was further purified via preparative RP-HPLC (Phenomenex Hyperclone 5 µ) using methanol/acetonitrile/water 85:6:9 containing 0.1% formic acid (flow 9 mL/min). Plakortide E methyl ester eluted at 14 min. Colourless viscous oil (3.2 mg, 33%); ${\left[\alpha \right]}_{D}^{23}$ = +74.1 (*c* 0.00305, CHCl_3_); LC-MS: *m*/*z* 403.9 [M + K]^+^, calcd. for C_22_H_36_O_4_ *m*/*z* 364.52. ^1^H and ^13^C NMR data were identical in all respects to those previously reported in the literature [40,41].

6-Epi-Plakortide H (**7**): The fraction containing several acids (**1mix**) was converted into an ester mixture (**7mix**) using the Steglich esterification procedure as described above for plakortide E methyl ester (**6**). Then, the mixture was purified using preparative RP-HPLC to yield the pure ester (**7**) which eluted at 14 min. Pale yellow viscous oil (6.6 mg), ${\left[\alpha \right]}_{D}^{23}$ = −107.14 (*c* 0.0028, CHCl_3_) [53] (see in the reference plakortide H methyl ester, ${\left[\alpha \right]}_{D}^{20}$ = +5.5 (*c* 2.9, CHCl_3_), 4-epi-plakortide H methyl ester ${\left[\alpha \right]}_{D}^{20}$ = +19 (*c* 0.13, CHCl_3_)). LC-MS: *m/z* 389.1 [M + Na]^+^, calcd. for C_22_H_38_O_4_ *m/z* 366.53. The absolute configuration was assigned as 6*R*, 10*R* in analogy with that of the 6-epi-plakortide H acid (**1**).

### 4.2. Cytotoxicity Assays

The origin and the maintenance of the cell lines were reported previously [45,46,47]. The resazurin reduction assay [43] was performed to determine the cytotoxicity of the seven compounds in a concentration range of 0.001 to 10 µg/mL as previously described [47,48].

## Figures and Tables

**Table 1:** ^1^H Nuclear magnetic resonance NMR (600 MHz), ^13^C NMR (150 MHz), and nuclear Overhauser exchange spectroscopy (NOESY) spectral data for 6-epi-plakortide H acid (**1**) in CDCl_3_.

Position	δ_C_	Mult	δ_H_	Mult	*J* in Hz	NOESY
1	177.06	C				
2	31.31	CH_2_	3.07 (2a)	dd	15.9, 9.6	2b, 5b
			2.41 (2b)	dd	15.9, 3.4	2a, 2b, 17
3	78.64	CH	4.44	ddd	3.3, 5.2, 9.5	2a, 2b, 4
4	35.37	CH	2.09 ^a^			3, 5a, 7
5	35.52	CH_2_	1.61 ^a^ (5a)	m		4, 7, 5b
			1.26 ^a^ (5b)			2a, 2b, 5a, 15
6	84.49	C				
7	127.13	CH	5.12	s		4, 5a, 9b
8	137.58	C				
9	47.60	CH_2_	2.06 ^a^–1.94 ^a^			7
10	42.60	CH	2.02 ^a^			
11	133.14	CH	5.09	dd	15.1	
12	131.89	CH	5.35	dt	15.1, 6.2, 6.2	
13	25.77	CH_2_	1.97 ^a^			
14	14.15	CH_3_	0.98	t	7.4	
15	32.58	CH_2_	1.55	m		5b
16	7.78	CH_3_	0.86	t	7.4	
17	25.12	CH_2_	1.16 ^a^			2b
18	11.12	CH_3_	0.92	t	7.6	
19	28.05	CH_2_	1.39			
			1.17 ^a^	m		
20	11.78	CH_3_	0.84	t	7.4	
21	17.04	CH_3_	1.70	s		

Chemical shift values are in ppm relative to the residual peaks of CDCl_3_ at 7.26 ppm (^1^H), and 77.16 ppm (^13^C). Spectra were recorded at 25 °C. ^a^ Overlap with other signals. For the methyl ester **7**, the same NOE correlations were found.

**Table 2:** Cytotoxicity of endoperoxides **1**–**7** and reference drugs against sensitive and multi-drug resistant leukemia cell lines.

Compound	CCRF-CEM IC_50_ [µM]	CEM/ADR5000 IC_50_ [µM]	Resistance Ratio
6-epi-Plakortide H acid (**1**)	0.18 ± 0.003	0.36 ± 0.01	2.00
Plakortide E (**2**)	1.90 ± 0.09	4.30 ± 0.1	2.26
Plakortin (**3**)	1.97 ± 0.06	2.26 ± 0.08	1.15
Dihydroplakortin (**4**)	1.13 ± 0.11	1.85 ± 0.13	1.64
Plakortic acid (**5**)	0.19 ± 0.004	0.24 ± 0.009	1.26
Plakortide E methyl ester (**6**)	NI ^1^	NI ^1^	N/A
6-epi-Plakortide H (**7**)	NI ^1^	NI ^1^	N/A
Doxorubicin *	0.012 ± 0.002	12.2 ± 54.2	1,036
Epirubicin *	0.022 ± 0.003	10.50 ± 3.90	484
Vincristine *	0.002 ± 0.0001	1.04 ± 0.15	613
Docetaxel *	0.0004 ± 0.0001	0.18 ± 0.02	438
Paclitaxel *	0.004 ± 0.0004	0.741 ± 0.137	200

^1^ NI, no inhibition at 27 µM; * data taken from reference [49].

## Supplementary Materials

The following are available online at www.mdpi.com/1660-3397/15/3/63/s1, Table S1: NMR data of plakortin (**3**); Table S2: Dihydroplakortin (**4**); Table S3: Plakortic acid (**5**); Table S4: Plakortide E methyl ester (**6**); Table S5: 6-epi-Plakortide H (methyl ester) (**7**); Figure S1: Structures of natural (**1**, **2**, **3**, **4**) and semi-synthetic (**5**, **6**, **7**) endoperoxides from a sample of the sponge *Plakortis halichondrioides*: 6-epi-plakortide H acid (**1**), plakortide E (**2**), plakortin (**3**), dihydroplakortin (**4**), plakortic acid (**5**), plakortide E methyl ester (**6**), and 6-epi-plakortide H (**7**).

## Abbreviations

DCC	dicyclohexylcarbodiimide
DMAP	4-dimethylamino pyridine
MDR	multi-drug resistant
NMR	nuclear magnetic resonance
RP-HPLC	reversed phase high performance liquid chromatography
SAR	structure-activity relationship
TLC	thin layer chromatography
