# Supplementary material for: Cytotoxicity of Endoperoxides from the Caribbean Sponge Plakortis halichondrioides towards Sensitive and Multidrug-Resistant Leukemia Cells: Acids vs. Esters Activity Evaluation
Source: Mar Drugs. 2017 Mar 3;15(3):63. doi: 10.3390/md15030063 (PMC5367020; doi:10.3390/md15030063)
Supplement: Supplementary file 1 [file marinedrugs-15-00063-s001.pdf]

Supplementary Materials: Cytotoxicity of endoperoxides from the Caribbean sponge Plakortis halichondrioides towards sensitive and multidrug-resistant leukemia cells: acids vs. esters activity evaluation

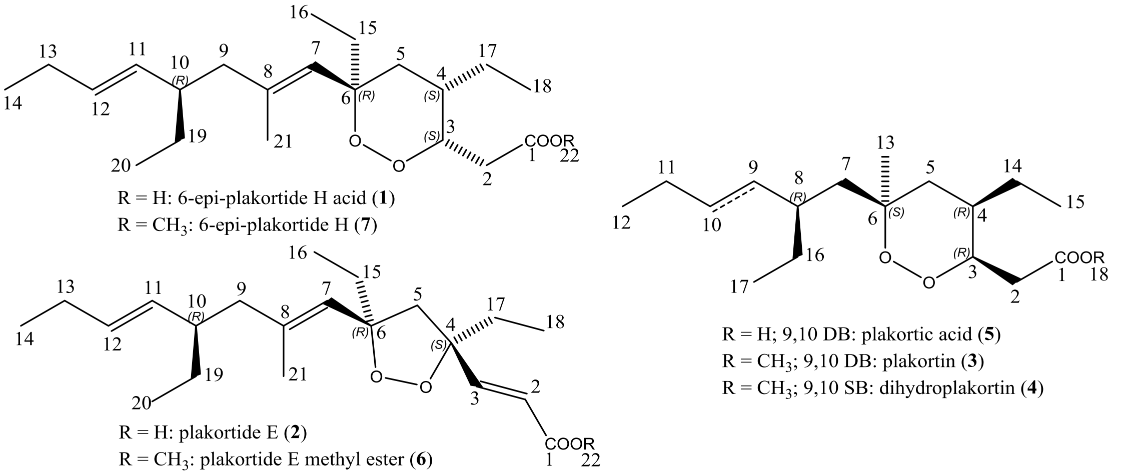

Figure S1: Structures of natural (**1**, **2**, **3**, **4**) and semi-synthetic (**5**, **6**, **7**) endoperoxides from a sample of the sponge *Plakortis halichondrioides*: 6-epi-plakortide H acid (**1**), plakortide E (**2**), plakortin (**3**), dihydroplakortin (**4**), plakortic acid (**5**), plakortide E methyl ester (**6**), and 6-epi-plakortide H (**7**).

Table S1: NMR data of plakortin (**3**)

Position	δ_C	mult	δ_H	mult	*J* in Hertz
1	172.45	C			
2	31.57	CH_2	3.01<br>2.36	dd<br>dd	15.6, 9.4<br>15.6, 3.6
3	79.12	CH	4.52 – 4.46	m	
4	34.87	CH	2.20 – 2.09	m	
5	36.10	CH_2	1.55<br>1.30[a]	dd	13.5, 4.4
6	81.50	C			
7	46.56	CH_2	1.47	dd	14.1, 4.2
8	40.34	CH	2.09 – 1.95	m	
9	134.41	CH	5.11	dd	15.3, 9.1
10	131.78	CH	5.38	dt	15.3, 6.3
11	25.67	CH_2	2.09 – 1.95	m	

12	14.03	CH_3	0.97	t	7.5
13	21.37	CH_3	1.37	s	
14	25.26	CH_2	1.21[a]<br>1.08[a]		
15	11.20	CH_3	0.90	t	7.4
16	30.06	CH_2	1.42[a]<br>1.15[a]	m<br>m	
17	11.62	CH_3	0.81	t	7.4
18	51.94	CH_3	3.70	s	

NMR solvent: $CDCl_3$; [a]overlap with other signals. Mult: multiplicity

Table S2: Dihydroplakortin (**4**)

Position	δ_C	mult	δ_H	mult	*J* in Hertz
1	172.38	C			
2	31.52	CH_2	3.01<br>2.37	dd<br>dd	3.66, 15.53<br>9.55, 15.52
3	78.75	CH	4.51	m	
4	34.94	CH	2.20	m	
5	35.42	CH_2	1.45<br>1.33[a]	ddt	5.35, 5.35, 10.62, 15.07
6	81.47	C			
7	45.48	CH_2	1.35[a]		
8	34.13	CH	1.45		
9	34.56	CH_2	1.24[a]		
10	29.03	CH_2	1.23[a]		
11	23.19	CH_2	1.29[a] –1.23[a]		
12	14.32	CH_3	0.89	m	
13	21.54	CH_3	1.37	s	
14	25.28	CH_2	1.22[a] –1.15[a]		
15	11.21	CH_3	0.92	t	7.96
16	27.45	CH_2	1.34[a] –1.29[a]		
17	10.74	CH_3	0.82	td	4.91, 8.49, 7.40
18	51.99	CH_3	3.71	s	

NMR solvent: $CDCl_3$; [a] overlap with other signals.

Table S3: Plakortic acid (**5**)

Position	δ_C	mult	δ_H	mult	*J* in Hertz
1	175.45	C			
2	31.57	CH_2	3.05<br>2.40	dd<br>dd	15.8, 9.6<br>15.8, 3.5
3	78.63	CH	4.52 – 4.46	m	
4	34.62	CH	2.25 – 2.09	tdd	12.6,7.3,5.0

5	35.67	CH_2	1.55<br>1.34[a]	dd	13.5, 4.4
6	81.50	C			
7	46.33	CH_2	1.47	m	
8	40.16	CH	2.09[a] – 1.95[a]		
9	134.15	CH	5.11	ddt	15.3,9.1,1.3
10	131.65	CH	5.39	dt	15.3, 6.3
11	25.46	CH_2	2.09[a] – 1.95[a]		
12	13.83	CH_3	0.97	t	7.5
13	21.25	CH_3	1.38	s	
14	25.00	CH_2	1.21[a]<br>1.08[a]		
15	10.96	CH_3	0.90	t	7.3
16	29.86	CH_2	1.42[a] – 1.15[a]		
17	11.42	CH_3	0.81	t	7.4

NMR solvent: $CDCl_3$; [a]overlap with other signals.

Table S4: Plakortide E methyl ester (**6**)

Position	δ_C	mult	δ_H	mult	*J* in Hertz
1	167.18	C			
2	120.03	CH	6.08	d	15.8
3	149.85	CH	6.86	d	15.8
4	87.32	C			
5	56.10	CH_2	2.54<br>2.44	d<br>d	12.0<br>12.0
6	89.39	C			
7	126.81	CH	5.12	s	
8	136.82	C			
9	46.65	CH_2	1.97<br>1.86	m<br>m	
10	42.66	CH	1.98	m	
11	132.91	CH	5.07	dd	15.2,8.2
12	132.07	CH	5.35	m	
13	25.72	CH_2	1.95	m	
14	14.20	CH_3	0.93	m	
15	32.33	CH_2	1.88<br>1.63	m<br>m	

16	9.03	CH_3	0.87	m	
17	30.97	CH_2	1.76	m	
18	8.97	CH_3	0.88	m	
19	27.73	CH_2	1.35<br>1.09	m<br>m	
20	11.72	CH_3	0.81	t	7.3
21	17.91	CH_3	1.61	d	1.0
22	51.73	CH_3	3.74	s	

NMR solvent: $CDCl_3$

Table S5: 6-epi-Plakortide H (methyl ester) (**7**)

Positions	δ_C	mult	δ_H	mult	*J* in Hertz
1	172.53	C			
2	31.44	CH2	3.04<br>2.38	dd<br>dd	15.8, 9.4<br>15.8, 3.4
3	78.90	CH	4.46	ddd	2.2, 4.3, 5.51
4	35.43	CH	2.09[a]		
5	35.53	CH_2	1.69[a] –1.20[a]	m	
6	84.34	C			
7	127.34	CH	5.13	s	
8	137.32	C			
9	47.60	CH_2	2.06[a] – 1.94[a]		
10	42.60	CH	2.02[a]		
11	133.14	CH	5.08	m	
12	131.87	CH	5.37	dt	15.1, 6.2, 6.2
13	25.77	CH_2	1.97[a]		
14	14.15	CH_3	0.98	t	7.4
15	32.56	CH_2	1.55	m	
16	7.78	CH_3	0.86	t	7.4

17	25.12	CH_2	1.17[a]		
18	11.15	CH_3	0.92	t	7.6
19	28.05	CH_2	1.40<br>1.18[a]	m	
20	11.78	CH_3	0.84	t	7.4
21	17.03	CH_3	1.70	d	
22	52.01	CH_3	3.71	s	

NMR solvent: $CDCl_3$; [a]overlap with other signals.
